# A Preliminary Assessment of Short-Term Social and Substance Use-Related Outcomes Among Clients of Integrated Safer Opioid Supply Pilot Programs in Toronto, Canada

**DOI:** 10.1007/s11469-023-01219-3

**Published:** 2023-12-22

**Authors:** Frishta Nafeh, Tinkhani Mbichila, Zachary Bouck, Ayden Scheim, Sanjana Mitra, Matthew Bonn, Franky Morris, Kate Atkinson, Kate Mason, Jolene Eeuwes, Carol Strike, Tara Gomes, Dan Werb, Mohammad Karamouzian

**Affiliations:** 1 Centre On Drug Policy Evaluation, MAP Centre for Urban Health Solutions, St. Michael’s Hospital, Toronto, ON Canada; 2 Dalla Lana School of Public Health, University of Toronto, Toronto, ON Canada; 3 Department of Epidemiology and Biostatistics, Dornsife School of Public Health, Drexel University, Philadelphia, PA USA; 4 Canadian Association of People Who Use Drugs, Dartmouth, NS Canada; 5 Street Health, Toronto, ON Canada; 6 Parkdale Queen West Community Health Centre, Toronto, ON Canada; 7 South Riverdale Community Health Centre, Toronto, ON Canada; 8 Li Ka Shing Knowledge Institute, St. Michael’s Hospital, Toronto, ON Canada; 9 Leslie Dan Faculty of Pharmacy, University of Toronto, Toronto, ON Canada; 10 Division of Infectious Diseases and Global Public Health, University of California San Diego, La Jolla, CA USA

**Keywords:** Safer Supply, Overdose, Harm Reduction, Canada

## Abstract

Canada is experiencing an ongoing overdose crisis, driven by a toxic unregulated drug supply. Integrated safer supply pilot programs offer pharmaceutical alternatives, coupled with comprehensive support services, to individuals using unregulated drug supply who are at high risk of overdose. We collected data from December 2020 to January 2023 on clients receiving safer opioid supply from five frontline service providers in Toronto, Canada, using interviewer-administered questionnaires. We assessed the incidence rate ratio of self-reported overdose comparing pre- and post-enrolment in the programs and examined changes in the prevalence of social and substance use outcomes post-enrolment. Forty-one participants were recruited, of whom 26 were followed up for a median of eight months (interquartile range, 5.0–11.7). The incidence rate ratio of overdose comparing post-enrolment to pre-enrolment was 0.20 (95% confidence interval, 0.09–0.43). Participants reported several positive social and substance use outcomes at follow-up, including a reduction in reliance on the unregulated supply and reduced criminal activity. Future implementation of integrated safer opioid supply pilot programs with larger sample sizes and rigorous epidemiological designs could help further illustrate the potential impacts of these programs in reducing overdose rates in Canada.

**Supplementary Information:**

The online version contains supplementary material available at 10.1007/s11469-023-01219-3.

Canada continues to grapple with a deadly overdose crisis driven by an unregulated drug supply contaminated with highly potent and toxic synthetic opioids (e.g., fentanyl and its analogues) and non-opioid substances, such as stimulants and benzodiazepines (Government of Canada, 2023a). In response, several policies and programs at both federal and provincial levels have been put into effect to circumvent growing drug related harms, including increased access to opioid agonist therapy, supervised consumption services, overdose prevention sites, naloxone distribution programs, and drug checking services (Strike & Watson, 2019). While these strategies have prevented thousands of overdose deaths (Irvine et al., 2019), they have not been able to address the toxic unregulated drug supply that is driving the overdose crisis (Government of Canada, 2023a), therefore creating a need for provision of safer substitutes to unregulated drugs.

Safer supply is an umbrella term referring to prescribing unsupervised doses of pharmaceutical alternatives to the unregulated drug supply (Glegg et al., 2022). Although the first pilot program providing safer opioid supply (SOS) was introduced in London, Ontario in 2016 (Gomes et al., 2022), the implementation of additional SOS pilot programs across multiple Canadian regions began in earnest in 2020, primarily via federal funding from Health Canada (Glegg et al., 2022). These pilot programs employ a variety of safer supply delivery models integrated within existing community clinical and social services (Karamouzian et al., 2023). Toronto, the largest metropolitan area in Canada, has experienced a drastic rise in opioid poisoning deaths in recent years. In 2022, the number of opioid-related deaths was 71% higher than in 2019 and 172% higher than in 2016 (Toronto Public Health, 2023). In response to the unabated opioid overdose crisis in Toronto, several SOS pilot programs were developed, co-located within existing community-based health centers or harm reduction programs and integrated with available wrap-around care programs across its downtown (Government of Canada, 2023b).

Emerging data on pilot safer supply programs with various delivery models and settings show promising outcomes including improvements in health and well-being, decline in overdose risk, and reduction in use of unregulated drugs (Bardwell et al., 2023; Gomes et al., 2022; Ivsins et al., 2021; Lew et al., 2022; McNeil et al., 2022). The available evidence on these pilot programs is, however, predominantly qualitative and there are limited quantitative findings regarding the effect of program enrolment on health outcomes among people who use drugs (Gomes et al., 2022; Lew et al., 2022). To address this knowledge gap, this study presents a preliminary assessment of overdose, as well as social and substance use-related outcomes among people who use SOS pilot programs in Toronto, Canada.

## Methods

### Study Setting and Design

SOS pilot program clients included in this study received take-home, short acting hydromorphone tablets with or without a long-acting “backbone” formulation, such as slow-release oral morphine or methadone, at one of the five integrated SOS programs in Toronto. Clients also received support with case management, appointment accompaniment, counseling, harm reduction education, recreational/drop-in programming, mobile care, and connection to other services in the community. Clients in these SOS pilot programs are required to follow-up with care on a regular basis, the frequency of which depends on the agreement with their care team.

The minimum requirement to enroll in pilot programs at these sites is daily or near-daily use of unregulated opioids at intake, confirmed via urinalysis. Each participant is assessed by the team to identify urgency and priority based on additional criteria (e.g., medical issues from injection drug use, recent history of overdose, homelessness, and racial and gender minority status) to prioritize individuals at high risk of opioid-related harms. Subsequently, program clients have their SOS doses titrated at the centers under the care of a nurse practitioner, and subsequently, they collect their medication from a pharmacy of their choice.

For the present study, SOS pilot program clients were recruited from the Toronto Disparities, Overdose and Treatment (T-DOT) study, a longitudinal observational cohort study of people who use drugs (Scheim et al., 2021) in Toronto, Ontario. T-DOT participants are adult Toronto residents, who can speak and understand English, and report past-6-month use of drugs other than cannabis, alcohol and tobacco. In this interim analysis, we used data collected between December 2020 through January 2023 on SOS pilot program clients from the five frontline service providers in Toronto.

Consenting participants completed a pilot-tested, interviewer-administered, survey questionnaire at baseline, which included items about participants’ sociodemographic characteristics, sociostructural exposures, and drug use behaviour. Those who completed a survey at baseline were invited to complete follow-up surveys at 1 month, 3 months, 6 months, and then every 6 months thereafter, which included items about participants’ sociodemographic, sociostructural exposures, drug use behaviour, and experience being on the pilot programs. Consent forms included information about the study, conditions for participation, potential risks and benefits, and details regarding ensuring confidentiality and protecting anonymity of study participants. All study participants provided voluntary, written informed consent prior to their intake survey, and received a $30 honorarium for each study visit.

### Variables

The primary outcome was self-reported, non-fatal overdose, assessed at baseline (i.e., before enrolment in SOS) and during follow-up surveys (i.e., post-enrolment in SOS). At baseline, participants were asked to report number of overdoses they experienced within the past six months, whereas, at follow-up surveys, participants were asked to report number of overdoses they had since their last follow-up interview. Sociodemographic variables included age, gender, self-identified race, employment, education, and current or recent diagnosis of comorbid mental and physical illness. Mental and physical illness were measured using multiple choice questions (Table 1). Sociostructural exposures included self-reported lifetime history of incarceration and homelessness, defined as having lived on the streets, parks, cars, or abandoned buildings during the past six months. Drug use behaviours included self-reported daily injection and non-injection use of fentanyl, heroin, and methamphetamine, powder cocaine, and crack cocaine.

**Table 1:** Characteristics of study sample at baseline (*n* = 41)

Characteristics	*n* (%)
Age (median)	39 (IQR^a^ = 32–45)
Gender	
Men	21 (51.2)
Women	20 (48.8)
Race^b^	
White	16 (59.3)
First Nation/Inuit/Metis	10 (37.0)
Black African/Caribbean	1 (3.70)
Homelessness^c^	
Yes	14 (34.1)
No	27 (65.9)
Full-time job	
Yes	1 (2.4)
No	40 (97.6)
Income sources in last 6 months^d^	
Social assistance	20 (48.7)
Legal street hustling^e^	11 (26.8)
Family support	4 (9.7)
Drug dealing	3 (7.3)
Employment	3 (7.3)
Sex work	2 (4.8)
Other	8 (19.5)
Education	
High school and above	26 (63.4)
Less than high school	15 (36.6)
Ever incarcerated	
Yes	13 (31.7)
No	28 (68.3)
Mental illness comorbidities^d^	
Post-traumatic stress disorder	20 (48.8)
Other mental illnesses^f^	9 (22.0)
Physical health comorbidities^d^	
Hepatitis C	12 (29.3)
Chronic obstructive pulmonary disease	3 (7.3)
Diabetes	3 (7.3)
Heart disease	2 (4.9)
Daily injection drug use (self-report)	
Fentanyl	23 (56.1)
Methamphetamine	4 (9.8)
Heroin	2 (4.9)
Powder cocaine	1 (2.4)
Crack cocaine	0 (0.0)
Daily non-injection drug use (self-reported)	
Fentanyl	5 (12.2)
Methamphetamine	3 (7.3)
Heroin	0 (0.0)
Powder cocaine	0 (0.0)
Crack cocaine	2 (4.9)

^a^*IQR:* interquartile range

^b^Only 27 participants out of the total sample (*n* = 41) provided a response

^c^Homelessness was defined as living on the streets, parks, cars, or abandoned buildings during the past six months

^d^Participants could select multiple options

^e^Legal street hustling included recycling, panhandling, and window cleaning (i.e., squeegeeing)

^f^Other mental illnesses refers to bipolar disorder, borderline disorder, major aggressive disorder, general anxiety disorder, depression, obsessive–compulsive disorder, anxiety, or body dysmorphia

Changes in social and substance use related outcomes were measured by asking study participants “*How has enrolment in the SOS program helped you?*” (Check all that apply): Reduced or no spending on unregulated drugs, stopped engagement in criminalized forms of income generation (e.g., theft, drug dealing, shoplifting), stopped being arrested and/or going to jail, improved food security (i.e., availability of a sufficient supply of reasonably priced and nutritious food), improved housing (i.e., any positive change in housing situation), helped cut down on unregulated opioid use, helped cut down or quit using other drugs (e.g., stimulants, alcohol), overall health improved, helped quit using unregulated opioids, got paid work, went to school, or got retrained. Study participants were also asked to rate their satisfaction with SOS using a Likert scale.

### Analysis

Descriptive statistics were used to summarize the sociodemographic, sociostructural exposures, and injection and non-injection drug use characteristics of study participants at baseline and their last follow-up visit. Incidence rate ratio (IRR) comparing overdose rates during the follow-up period to the baseline was estimated, and 95% confidence intervals (CI) were reported using a negative binomial generalized estimation equation regression model to account for overdispersion and an exchangeable correlation structure to account for the correlated nature of the data (Lawless, 1987; Yirga et al., 2020). We compared the baseline characteristics of participants lost to follow-up with those who had at least one follow-up visit using the chi-squared and Fisher’s exact tests. We compared daily injection drug use across four drugs using McNemar’s test. *P* values < 0.05 were considered to indicate statistical significance.

We conducted sensitivity analyses to assess the potential impact of loss to follow-up on the overdose incidence rate estimates. We explored different worst-case scenarios among those lost to follow-up by assuming 1, 2, 3 or 4 overdose incidents per person during a comparable hypothetical median follow-up period to those who had at least one follow-up visit. The range of presumed overdoses in the sensitivity analysis was informed by the median number of overdoses at baseline among the individuals who were lost to follow-up (i.e., 1). Data analysis was performed using SAS version 9.04 and Stata version 16.

## Results

### Participant Characteristics

Of the total 41 adult SOS clients recruited into this study, 15 were lost to follow-up (they did not complete any follow-up surveys after completion of the baseline survey); the remaining 26 (63%) were followed for a median of 8.5 months (interquartile range [IQR] = 5.0–11.7). Table 1 summarizes sociodemographic characteristics, sociostructural exposures, and daily injection and non-injection drug use behaviour of study participants at baseline. Participant median age was 39 years (IQR = 32–45), and over half of the study participants self-reported their race as White (*n* = 16; 59.3%) and self-identified as men (*n* = 21; 51.2%). About half (*n* = 20; 48.7%) relied on social assistance as a source of income in the previous six months and only one person reported having a full-time job. Nearly half reported a current diagnosis of post-traumatic stress disorder (*n* = 20; 48.8%), and one-fifth reported a current diagnosis of other mental illness (*n* = 9; 22.0%). In terms of physical health comorbidities, current diagnosis of hepatitis C was the most reported condition (*n* = 12; 29.3%). Fentanyl was the most injected drug (*n* = 23; 56.1%) and non-injected drug (*n* = 5; 12.2%). There were no statistically significant differences between baseline characteristics, including baseline rate of self-reported non-fatal overdose of participants retained in the study compared to those lost to follow-up (Supplementary Table 1).

### Overdose Incidence

Among the 26 individuals with follow-up visits, there were 15 self-reported overdose incidents during the 235 person-month follow-up period (IR = 7.06 per 100 person-months; 95% CI, 3.35–14.82) and 57 self-reported overdose incidents in the six-month period prior to enrolment in the program corresponding to 156 person-months (IR = 35.16 per 100 person-months; 95% CI, 14.87–83.52). The IRR of overdose comparing follow-up to pre-enrolment (i.e., baseline) adjusted for age and gender was 0.20 (95% CI, 0.09–0.43). The findings remained stable in the sensitivity analyses assuming 1 to 4 overdose events among those lost to follow-up (Supplementary Table 2).

### Social and Substance Use Outcomes Post-enrolment in the Pilot Programs

Over half of the participants (*n* = 15; 57.7%) reported excellent satisfaction with the pilot programs. At their last follow-up visit, many participants self-reported a reduction or no spending on unregulated drugs (*n* = 14; 53.8%), stopped engagement in criminalized forms of income generation (n = 13; 50.0%), stopped being arrested or going to jail (*n* = 12; 46.2%), and reported improvement in food security (*n* = 12; 46.2%). One-third (*n* = 9; 34.6%) of the  participants reported improvement in their housing situation. With regard to substance use-related outcomes, participants reported a reduction in the amount of their unregulated opioid use (*n* = 16; 61.5%) and cutting down or stopping their use of other drugs (*n* = 16; 61.5%). Furthermore, half of the participants reported an improvement in their overall health (*n* = 13; 50.0%). Three (11.5%) reported having quit using unregulated opioids since receiving SOS, 3 (11.5%) found paid work and 2 (7.7%) went to school/retrained. There were no statistically significant change (increase or decrease) in participants’ self-reported daily injection or non-injection use of fentanyl, heroin, methamphetamine, powder cocaine, and crack cocaine after enrolment in the pilot programs (*P* values > 0.05).

## Discussion

We followed 26 SOS pilot program clients, from 2020 to 2022 for a median of 8.5 months and found that the rate of overdose during the follow-up period (i.e., enrolment in a pilot program) was 0.20 times their rate of overdose during the pre-enrolment period. Although our findings are preliminary and the study sample size is small, the sensitivity analyses (i.e., assuming 1 to 4 overdose incidents among those lost to follow-up) showed a stable incidence rate.

Our preliminary findings are encouraging and in line with the growing body of evidence on pilot SOS programs. For example, a previous study of a SOS program operated within an emergency shelter in Hamilton, Ontario, also reported reduced incidence of non-fatal overdose. Specifically, the odds of experiencing a non-fatal overdose were found to be 5.5 times higher before the program was established compared to after its operation (Lew et al., 2022). A matched cohort study from London, Ontario, also showed a significant reduction in emergency department visits and hospital admissions after engagement with a pilot SOS program (Gomes et al., 2022). Additionally, several qualitative studies have reported a self-perceived reduction in participants’ overdose risk after program enrolment (Bardwell et al., 2023; Health Canada, 2022; Ivsins et al., 2021; Kolla et al., 2022; McNeil et al., 2022) as well as improvement in general health (Bardwell et al., 2023; Health Canada, 2022; Ivsins et al., 2021; Kolla et al., 2022).

Consistent with the existing qualitative studies from Canada that have reported decreased use of unregulated drugs among safer supply clients (Bardwell et al., 2023; Health Canada, 2022; Ivsins et al., 2021; Ivsins et al., 2022), we noted that some participants self-reported a reduction in use of unregulated opioids and other drugs after enrolment in SOS. However, about 40% still relied on unregulated opioid supply and daily injection or non-injection drug use did not decrease significantly. This could be due to inadequate dosing of available medications that fail to match the current strength of highly potent, street fentanyl and therefore cannot sufficiently manage withdrawal among individuals who have high tolerance to these drugs (Bardwell et al., 2023; Karamouzian et al., 2023).

Our preliminary findings regarding reduced structural vulnerability, such as improved housing, reduced engagement in criminalized activities, and interactions with law enforcement, are encouraging. They are also consistent with qualitative evaluations of pilot programs conducted in Ontario and British Columbia. These studies have reported a reduction in criminalized forms of income generation (Ivsins et al., 2022), reduced engagement with unregulated drug markets (McNeil et al., 2022), and improvement in overall well-being among program clients (Bardwell et al., 2023; Ivsins et al., 2021). Our study also showed self-reported improvements in housing and food security consistent with the findings of Health Canada’s preliminary qualitative assessment, conducted between December 2020 to March 2021 (Health Canada, 2022). These benefits appear due to the additional benefits of integrating wrap-around health and social service into safer supply programs (Government of Canada, 2023b; Glegg et al., 2022; Health Canada, 2022).

## Limitations

Given the limitations of this preliminary study, it is important to interpret the findings with caution. First, our sample size was a small group of SOS clients with a high risk of overdose at baseline, and we used longitudinal regression analysis to estimate effect size. As such, we cannot confidently infer causality. Future studies would benefit from larger sample sizes and inclusion of a control cohort group not exposed to SOS. Second, there was substantial loss to follow-up of participants (37%) upon completion of baseline assessment as clients typically belong to highly marginalized groups and are therefore hard to reach. While the baseline characteristics, including baseline rate of overdose, of those lost to follow-up did not significantly differ from those who remained in the study and we undertook a sensitivity analysis to determine the likely range of the IRRs of self-reported overdose events prior to and after program enrolment, there remains considerable uncertainty. Third, although our sample initially contained roughly equal proportions of men and women, a higher proportion of women were lost to follow-up compared to men and we were unable to conduct gender-stratified analyses. The higher dropout rate among women and the established gendered nature of accessing and responding to substance use treatment interventions (Polak et al., 2015) highlights that gender-based differences in program retention are important features to investigate in future studies examining SOS programs. Fourth, our sample participants were predominantly White and we were unable to examine potential race-based differences in accessing care, which are well-documented in addiction medicine (Lewis et al., 2017; Mendoza et al., 2019). Fifth, our results are based on self-report and are subject to reporting and social desirability biases. Lastly, our findings are not generalizable to other settings or models of SOS delivery available in Canada.

## Conclusions

Following enrolment in pilot programs in Toronto, we observed that the overdose incidence rate appeared to be lower during follow-up among participants retained in our study. Many participants reported a number of positive social and substance use-related outcomes, including reduced reliance on the unregulated drug supply, reduced engagement in criminal activities, reduced interactions with law enforcement, and improved food security and housing status.  Most participants also reported being satisfied with the programs. These findings, however, are preliminary and need to be validated with more rigorous population-based studies using large sample size and control groups. Moreover, exploring program retention and clients’ engagement experience with pilot safer supply programs is an important area for future research, given that a notable proportion of participants were lost to follow-up in our sample. This is critical to determining the potential impacts of integrated safer supply programs in reducing harms among people who use drugs in Canada.

## Supplementary Information

Below is the link to the electronic supplementary material.

Supplementary file1 (DOCX 22.5 KB)

## Data Availability

The supporting data for the findings can be obtained upon reasonable request, as they are subject to confidentiality and ethics restrictions. Due to the small sample size and the presence of individual-level information that could potentially identify participants, the data cannot be publicly accessed.
