# Supplementary material for: A Preliminary Assessment of Short-Term Social and Substance Use-Related Outcomes Among Clients of Integrated Safer Opioid Supply Pilot Programs in Toronto, Canada
Source: Int J Ment Health Addict. 2023 Dec 22;23(3):2105–15. doi: 10.1007/s11469-023-01219-3 (PMC12279591; doi:10.1007/s11469-023-01219-3)
Supplement: Supplementary file 1 — Supplementary file1 (DOCX 22.5 KB) [file 11469_2023_1219_MOESM1_ESM.docx]

**Supplementary Table 1. Baseline characteristics of participants retained compared to those lost to follow-up**

| **Characteristics** | **Retained^a^**  **n=26 (%)** | **Lost to Follow-up**  **n=15 (%)** | **P-value^b^** |
| --- | --- | --- | --- |
| **Age (median)** | 41 (IQR^c^=33-46) | 34 (IQR=30-41) | 0.22 |
| **Gender** |  |  |  |
| *Men* | 15 (57.7) | 6 (40) | 0.28 |
| *Women* | 11 (42.3) | 9 (60) |  |
| **Race^d^** |  |  |  |
| *White* | 11 (42.3) | 5 (33.3) | 0.60 |
| *First Nations/Inuit/Metis* | 7 (26.9) | 3 (20) |  |
| *Black African/Caribbean* | 0 (0.0) | 1 (6.7) |  |
| **Mental illness comorbidities^e^** |  |  |  |
| *Post-traumatic stress disorder* | 11 (42.3) | 9 (60) | 0.34 |
| *Other mental illnesses^f^* | 5 (19.2) | 4 (26.7) | 0.70 |
| **Physical health comorbidities^e^** |  |  |  |
| *Hepatitis C* | 6 (23.1) | 6 (40) | 0.25 |
| *Chronic obstructive pulmonary disease* | 3 (11.5) | 0 (0.0) | 0.29 |
| *Diabetes* | 3 (11.5) | 0 (0.0) | 0.29 |
| *Heart disease* | 1 (3.8) | 1 (6.7) | 1.00 |
| **Daily injection drug use (self-report)** |  |  |  |
| *Fentanyl* | 17 (65.4) | 6 (40) | 0.12 |
| *Methamphetamine* | 7 (26.9) | 3 (20) | 0.72 |
| *Heroin* | 1 (3.8) | 1 (6.7) | 1.00 |
| *Powder cocaine* | 2 (7.7) | 1 (6.7) | 1.00 |
| *Crack cocaine* | 0 (0.0) | 0 (0.0) | 1.00 |
| **Daily non-injection drug use (self-reported)** |  |  |  |
| *Fentanyl* | 1 (3.8 ) | 3 (20.0) | 0.13 |
| *Methamphetamine* | 2 (7.7 ) | 1 (6.7) | 1.00 |
| *Heroin* | 0 (0.0) | 0 (0.0) | 1.00 |
| *Powder cocaine* | 0 (0.0) | 0 (0.0) | 1.00 |
| *Crack cocaine* | 2 (7.7) | 0 ( 0.0) | 0.52 |
| **Overdose rate in the last 6 months at baseline** | 36.6 per 100 person-months (95% CI: 11.5-116.0) | 32.2 per 100 person-months (96% CI: 13.6-76.5) | 0.84 |

^a^ Retained refers to study participants who had at least 1 follow-up visit and lost to follow-up refers to those with no follow-up interview.

^b^ Estimate obtained using Chi-Squared or Fisher’s Exact Test as appropriate.

^c^ IQR: Interquartile range.

^d^ Only 27 participants out of the total sample (n = 41) provided a response.

^e^ Participants could select multiple options.

^f^ Other mental illnesses refers to bipolar disorder, borderline disorder, major aggressive disorder, general anxiety disorder, depression, obsessive-compulsive disorder, anxiety, or body dysmorphia.

**Supplementary Table 2. Worse-case scenarios assuming those lost to follow-up remained in the study (n= 41)**

| **Hypothetical scenarios** | **Assumption^a^** | **Incidence rate ratio^b^ (95% CI)^c^** |
| --- | --- | --- |
| **Worse-case scenario 1** | *Median follow-up: 8.5 months;*  *1 overdose incident per person* | 0.26 (95% CI: 0.15 to 0.45) |
| **Worse-case scenario 2** | *Median follow-up: 8.5 months;*  *2 overdose incidents per person* | 0.38 (95% CI: 0.21 to 0.67) |
| **Worse-case scenario 3** | *Median follow-up: 8.5 months;*  *3 overdose incidents per person* | 0.49 (95% CI: 0.27 to 0.90) |
| **Worse-case scenario 4** | *Median follow-up: 8.5 months;*  *4 overdose incidents per person* | 0.60 (95% CI: 0.33 to 1.11) |

^a^ The assumed median follow-up time (i.e., 8.5 months) is similar to those remained in the study.

^b^ Incidence rate ratios are comparing the rate of overdose during the follow-up versus baseline, and are adjusted for age and gender of the participants.

^c^ CI: Confidence Intervals.
